# Laser-Induced Periodic Surface Structures and Their Application for Gas Sensing

**DOI:** 10.3390/mi15091161

**Published:** 2024-09-17

**Authors:** Johann Zehetner, Ivan Hotovy, Vlastimil Rehacek, Ivan Kostic, Miroslav Mikolasek, Dana Seyringer, Fadi Dohnal

**Affiliations:** 1 Research Centre for Microtechnology, Vorarlberg University of Applied Sciences (FHV), Hochschulstraße 1, 6850 Dornbirn, Austria; johann.zehetner@outlook.com (J.Z.); dana.seyringer@fhv.at (D.S.); fadi.dohnal@fhv.at (F.D.); 2 Institute of Electronics and Photonics, Slovak University of Technology, Ilkovicova 3, 812 19 Bratislava, Slovakia; vlastimil.rehacek@stuba.sk (V.R.); miroslav.mikolasek@stuba.sk (M.M.); 3 Institute of Informatics, Slovak Academy of Sciences, Dubravska cesta 9, 845 07 Bratislava, Slovakia; ivan.kostic@savba.sk

**Keywords:** laser ablation, laser-induced periodic surface structure, semiconducting metal oxides, optical gas sensing

## Abstract

Semiconducting metal oxides are widely used for solar cells, photo-catalysis, bio-active materials and gas sensors. Besides the material properties of the semiconductor being used, the specific surface topology of the sensors determines device performance. This study presents different approaches for increasing the sensing area of semiconducting metal oxide gas sensors. Micro- and nanopatterned laser-induced periodic surface structures (LIPSSs) are generated on silicon, Si/SiO_2_ and glass substrates. The surface morphologies of the fabricated samples are examined by FE SEM. We selected the nanostructuring and characterization of nanostructured source Ni/Au and Ti/Au films prepared on glass using laser ablation as the most suitable of the investigated approaches. Surface structures produced on glass by backside ablation provide 100 nm features with a high surface area; they are also transparent and have high resistivity. The value of the hydrogen sensitivity in the range concentrations from 100 to 500 ppm was recorded using transmittance measurements to be twice as great for the nanostructured target TiO_2_/Au as compared to the NiO/Au. It was found that such transparent materials present additional possibilities for producing optical gas sensors.

## 1. Introduction

Environmental issues, in particular global warming, have been moving researchers to focus on research in energy harvesting, energy storing and high energy conversion efficiency. In light of such topics of research, there is much interest in the effective usage of green energy. Among other things, this requires increases in electric capacitor and battery performance, and the development of new technologies for effective H_2_ generation on an industrial scale. Given this, gas sensor research is further gaining relevance, and to a great extent, tailored laser-induced micro- and nanotextured surfaces provide effective support for technological advancement. NiO and TiO_2_ are semiconducting metal oxides widely used for solar cells, photo-catalysis, bio-active material and H_2_-sensitive gas sensors. Besides the material properties of the semiconductor being used, the specific surface topology significantly influences device performance.

Sensor arrays incorporating multiple types of sensors with onboard data management lead to increasing data rates between the components. The progress of industrial printed wiring board technology (PWB) can offer one solution for this challenge in the combination of optical and electrical data lines in one PWB. Vertical cavity surface emitting lasers (VCSELs) are used as a transmitter and the optical waveguides are written by two-photon absorption (TPA) with a femtosecond laser in a polymer layer inside the PWB [1]. Moreover, the VCSELs can serve as a light source for optical sensors and integrated photonic elements used in optical data analyses [2]. Such an approach facilitates the combination and interaction of electro-chemical, photo-chemical, photonic and electronic sensor systems, and lab-on-chip applications. Surface functionalized templates in environmental [3], biological [4] and medical research and diagnostics [2] can become part of an advanced and intelligent network system. Recent progress in the wafer-scale production of metal oxide based on nanosensor arrays by photo pen writing [5] may also be very suitable for integration into next-generation nanotechnology systems. In [3,5], very cost-effective intense flashlight pulses and CO_2_ laser pulses were used, respectively, for metal oxide nanoparticle decoration on 3D microstructures and the fabrication of metal oxide nanostructured sensor arrays. Using a femtosecond laser system for nanoparticle generation cannot compete cost-wise with the previously mentioned methods, but it provides technological merits in the nano-scale and micrometer-scale structuring of functionalized surfaces as well. It is possible to produce black titanium dioxide decorated in situ on fs-laser-induced LIPSSs [6].

As mentioned above, surface properties are essential for sensor performance, and besides nanoparticles, LIPSSs are a proper method for manipulating them. Recent advancements in industrial laser technology have opened new applications in biology, medicine, optics, tribology, catalysis, photovoltaic, fuel cell research, etc. Many metals, semiconductors and dielectrics are suitable for LIPSS manufacturing. Consequently, in the present study, laser ablation was selected for contour shaping, surface patterning and surface functionalization. The phenomenon of LIPSS formation has already been a research topic for decades. However, only recently have the complex interactions of physical, photonic and electronic effects with specific material properties in solids become well understood. We give a brief overview of morphological variations in LIPSSs with respect to our application in electrical and optical gas sensor research and demonstrate the merits and drawbacks of different fabrication strategies. Our aim was to increase the surface area in gas sensor application and to generate gas-sensitive metal oxide nanoparticles. We comment on our experimental results in light of already existing comprehensive theoretical and experimental knowledge generated over the decades of LIPSS research. It is now accepted common knowledge to divide LIPSS into two main groups, low spatial frequency LIPSS (LSFL) with a spatial period of about the used laser wavelength, and high spatial frequency LIPSS (HSFL) with about half the period. For dielectrics, the spatial period can be reduced by the factor of the refractive index. A sub-classification is based on the orientation with respect to the laser polarization and a more specific period of the structure. Low laser fluency and a low number of pulses or consecutive scans facilitate the formation of HSFLs and increased numbers facilitate the formation of LSFL types [7,8]. The smoothness and regularity of LIPSS were found to be linked to the decay length of surface electromagnetic waves generated by the laser radiation. The regularity increases with a shorter decay length [9]. A new type of LSFL was found in recent research at the interface between the laser-induced oxide layer and the non-altered metal underneath [10]. Chemical effects in the formation of amorphous oxides on titanium alloys for tribological applications are researched in [11]. The formation chemistry may also be relevant in the generation process of amorphous nanoparticles. In our optical gas sensor research, we produced HSFL on glass by a backside ablation process using a metal interface directly placed at the glass surface on the sample backside. In a recent more detailed research, this method is named laser-induced plasma-assisted ablation (LIPAA). A variation in the LIPAA process utilizes interference-based laser-induced micro-plasma ablation of glass for complex surface texturing in the micrometer and nanometer range [12,13,14,15].

In the present study, laser ablation was selected for contour shaping, surface patterning and surface functionalization due to its all-purpose applicability for a great variety of standard materials and new advanced materials essential for specific applications. We give a brief overview of morphological variations in laser-induced periodic surface structures (LIPSS) and femtosecond (fs) laser-generated self-ordered hierarchical micro and nanostructures. We investigate the merits and drawbacks of different fabrication strategies in generating LIPSS to increase the surface area for gas sensor application.

## 2. Experimental Section

### 2.1. LIPSS Fabrication on Thermal Oxidized Si Wafer

**Figure 1:**
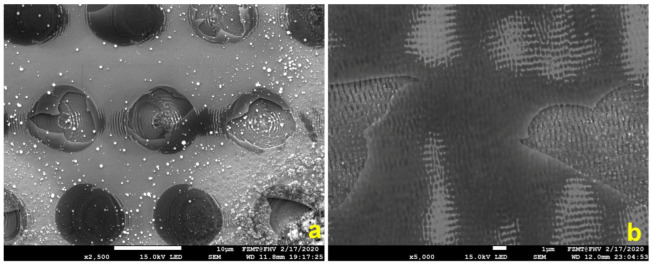
Failed attempt to generate LIPSS directly on a Si/SiO_2_ substrate (**a**) single pulse and (**b**) overlapping pulse procedure. LIPSS formation starts at the Si and not the SiO_2_ surface.

In the first approach, we intended to generate LIPSS directly on a Si/SiO_2_ wafer. However, the attempt failed. The wafer was thermally oxidized prior to the laser process and the oxide layer had a thickness of 350 nm. We could not successfully generate LIPSS directly on the Si/SiO_2_ substrate. Due to the significantly lower ablation threshold of Si with respect to SiO_2_, the laser structured area was chipping off the wafer already at single pulse exposure (Figure 1a). Unfortunately, increasing the pulse frequency to obtain a 50% overlap of the circular laser spots and reducing the laser power was not successful. The formation of LIPSS started at the Si surface below the SiO_2_ layer, and in some locations, the created plasma and shock wave removed the oxide (Figure 1b). We rejected this fabrication strategy and continued with an alternative method.

### 2.2. LIPSS Fabrication on Plane Si Wafer and Thermal Oxidation Experiment

**Figure 2:**
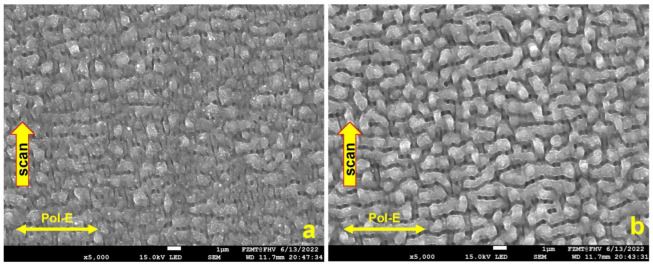
LIPSS fabrication directly on the Si wafer: (**a**) one scan generates LSFL-I type orientated perpendicular to polarization; (**b**) two scans cause transformation into LSFL-II type parallel orientated to polarization.

First, the LSFLs orientated parallel to the laser polarization were generated directly on a Si wafer, and in a second step, the oxide layer with a thickness of 350 nm was produced by thermal oxidation on top of the LSFLs. The laser wavelength was 520 nm, the laser pulse frequency was 1000 kHz, the pulse length was 350 fs, the scan speed was 1000 mm/s, the hatch between two scans was 5 µm, the focal length was 170 mm, the focus diameter was in the range of 12 to 14 µm and the measured average laser power was 456 mW. The result after one scan is shown in Figure 2a. LSFL-I-type structures perpendicular to the polarization begin to arise. However, one can already see additional, somewhat irregular structures. In the picture after the second scan (Figure 2b), one can see that the LSFL-I-dominated structures from the first scan, transformed to a great extent into LSFL-II-type structures, oriented parallel to the laser polarization. At any consecutive scan, the previously generated pattern interferes with laser radiation and contributes to metamorphoses toward a new pattern. Also, the number of scans and the laser fluence contribute to that transformation [7].

**Figure 3:**
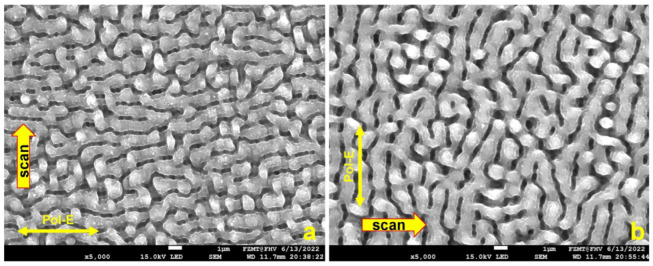
LIPSS fabrication directly on the Si wafer: (**a**) four scans and no changes from LSFL-II typ, only the structure becomes more distinct; (**b**) four scans with reversed polarization and scan direction, LSFL-II type parallel orientated to polarization but now in y direction.

**Figure 4:**
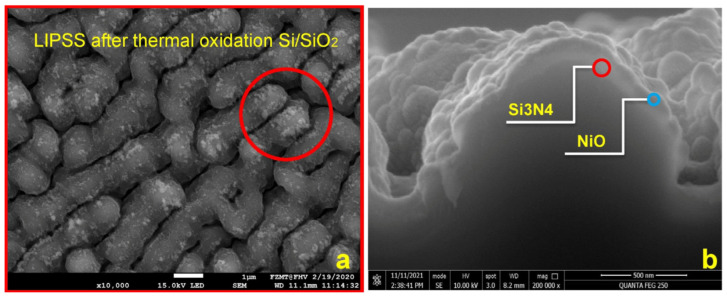
(**a**) Thermal oxide on LIPSS has poor insulating quality due to sintered debris (inside red circle) from ablation; (**b**) alternative solution was sputtered Si_3_N_4_ layer, red circle in the cross-section.

**Figure 5:**
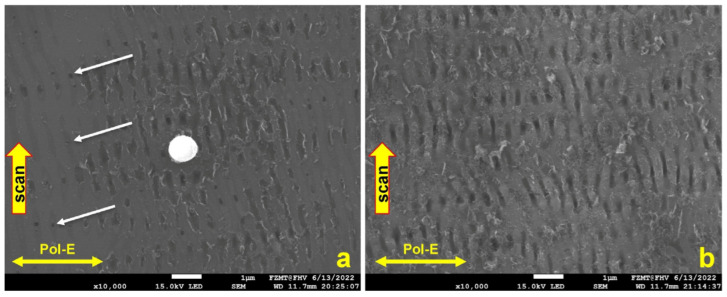
(**a**) Subsurface nonlinear ionization, first breaking through to the surface at the white aero-points after one scan; (**b**) skin-like debris from the ionization breaking through after 10 over-scans and LSFL-I formation phase started. Nano-plasma bubble defects in the Si bulk will be later confined in thermal oxide and reduce breakdown voltage.

Figure 3a shows that the LSFL-II-type structure did not convert into a new LIPSS type after 4 accumulated scans, but the LIPSS shape is slightly more distinct. In Figure 3b, the scan direction and polarization were reversed for all 4 scans; one can clearly see that the orientation of the LSFL-II structure is accordingly following the new laser polarization in agreement with [7]. Unfortunately, electrical measurements indicated that the thermal oxide had poor insulation quality. We assume that locally generated spongy structures with low ohmic resistivity and low electrical breakdown voltage are caused by ablation debris sintered onto the surface (Figure 4a; area inside the red circle on top of the thermal oxide). It is possible that an additional cause for the low resistivity is related to pre-surface-triggered nanoplasma explosions in the laser ablation process [16]. This might create nanobubble defects in the bulk close to the surface of LIPSS. After thermal oxidation, these nanobubbles are inside the thin insulation oxide layer causing a significantly reduced breakdown voltage. Figure 5 indicates that our second conclusion for the reduced breakdown voltage has a good possibility to be a real route cause. We used the same laser parameter as in the previous experiment, only the laser power was reduced to measured 329 mW. After one scan, the LIPSS formation just started, predominately at locations with particles causing a coherent field enhancement and facilitating LIPSS formation in their vicinity (Figure 5a, white sphere in the center, 1 µm in diameter). At a radial distance of about 3 to 4 µm, the ablation started pointwise (white arrows) and one gains the impression that LIPSS formation started just “under the skin” in a faint pattern of slightly darker parallel lines perpendicular to the laser polarization. According to [16], the surface ripple formation starts (state 1) with a subsurface nonlinear ionization of local defects, continues (state 2) with a periodic plasma sheet formation and finally ends (state 3) with breakdown and ablation of the surface. After 10 consecutive scans, state 3 is reached for nearly most of the ripples, but the process of distinct LIPSS formation is not yet finished. Still, very thin sheets of material (like peeling off sunburnt skin) are visible from the start of state 3 (Figure 5b). We believe this is a further indication that laser ablation and the LIPSS formation mechanism suffer from intrinsic issues for thin thermal oxide isolation fabrication. Figure 4b shows, as a consequence of this, the cross-section of a Si_3_N4 isolation layer on top of a single LSFL-II ripple for the final demonstrator version of a NiO-based electrical gas sensor [17].

### 2.3. LIPSS Generation on Glass by Metal-Supported Backside Ablation

**Figure 6:**
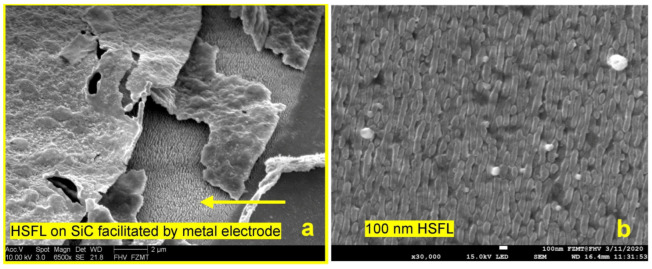
(**a**) Backside formation of HSFLs between a metal electrode and SiC sample surface due to LIPAA effect indicated by the yellow arrow; (**b**) 100 nm hatched (depicted by yellow line) HSFLs produced with LIPAA.

The third method we investigated was direct LIPSS generation on glass by backside ablation. Glass does not need to generate an isolation layer after the laser treatment. Moreover, it is a transparent material and can be used in electrical and optical sensor systems alike. In our previous research on laser-fabricated membranes in SiC for ferroelectric thin film MEMS [18], we observed that a metal interface at the backside of transparent dielectric materials and semiconductors reduces the ablation threshold and promotes the formation of HSFLs (Figure 6a) at a now-possible lower power setting [7]. The process was recently established as the LIPAA method and is well researched and developed [12,13,14,15]. We used a simple version of the procedure (confined laser plasma ablation (CLPA)) for selective mask writing to fabricate 3D micro-structures and nanoparticle-decorated LIPSS [6]. The laser light transmits the substrate and HSFLs are produced on the backside, facilitated by a metallic interface producing confined laser plasma at the glass surface. Nanoparticles formed and deposited at the glass in the first scan, and to some extent short wavelength components of the plasma, reduce the ablation threshold for the second scan. We show a demonstration of the enhanced ablation and LIPSS formation due to field effects in the vicinity of a nanoparticle in the results and discussion section. The lower laser power promotes the formation of grating-like HSFL in the 100 nm range (Figure 6b) [7]. For the sample in Figure 6b, we used an average laser power of 950 mW, a 1000 kHz laser pulse frequency, a 1000 mm/s scan speed in the y direction, a hatch of 5 µm, linear polarization in the x direction, a laser wavelength of 520 nm, a focal length of 170 mm, and two consecutive scans. No LIPSSs are generated at the front surface due to the considerable threshold reduction by the metal at the backside. We believe that in micro-optical sensor applications, HSFLs fit better to existing design opportunities for implementing optical gas sensors in polymer-based integrated photonic devices [11] and platforms. The coarse LSFL-II structure (Figure 4b) that we used in our electrical sensor demonstrator [17] can cause spatial light beam deflections to a scale not tolerable in alignment demands for micro-devices.

### 2.4. Metal Oxide Nanoparticle Films on Glass Produced by Confined Laser Plasma Ablation

**Figure 7:**
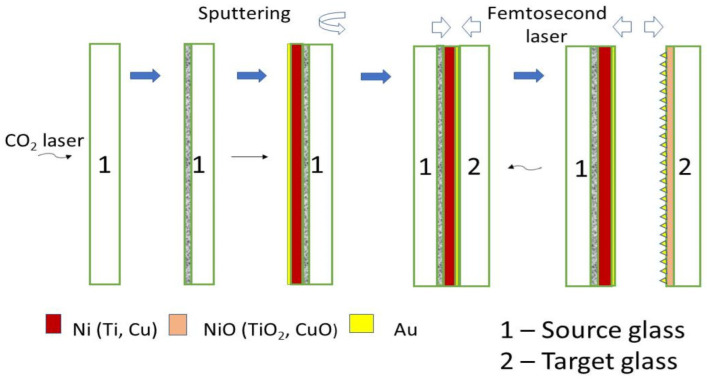
Preparation of LIPPS structures on microscopic glass.

The objective was to fabricate a glass substrate covered with a homogeneous thin NiO/Au or TiO_2_/Au composite film that was optically sensitive to hydrogen, methane and acetone. The effectiveness shall be tested in an optical gas sensor demonstrator as we proposed in [19]. This method resembles the first step of a LIPAA process. A source for Ni/Au and Ti/Au material is needed and a target is to collect the generated NiO/Au and TiO_2_/Au nanoparticles generated in the ablation and laser plasma process (see Figure 7). A microscope glass, its surface structured with a CO_2_ laser to generate micro cracks, was used as the source. After cleaning, the source slide was plasma-coated with 50 nm Ni or Ti and 10 nm Au, respectively. On top of the metal layer, a second microscope glass was placed, which was used as the target to collect the metal oxide nanoparticles. In the final step, the fs-laser light passes through the target glass substrate, ablates the metal coating at the source glass substrate and transforms it into metal oxide nanoparticles inside the laser plasma using the air inside the CO_2_-laser-generated cracks. Moreover, these cracks help to suppress light interference effects in the fs-laser transformation and the particle deposition process to homogenize the deposited film (without Newton fringes resembling film) is often the result. These nanoparticles (NiO/Au, TiO_2_/Au) are directly transferred out of the confined laser-generated plasma to the surface covering the glass plate, finally forming the gas sensor element. The laser parameters for the produced samples were set to an average laser power of 245 mW, at a 100 kHz laser pulse frequency, 500 mm/s scan speed in the x direction, a hatch of 5 µm, linear polarization in the x direction, a laser wavelength of 520 nm, a focal length of 170 mm, and one single scan.

**Figure 8:**
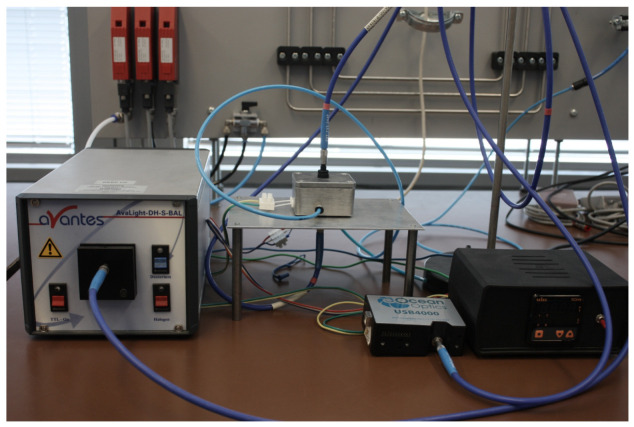
Experimental setup for optical gas sensing measurements.

The surface morphology and the elemental distribution were also observed by an FEI XL30 scanning electron microscopy (SEM) with an energy-dispersive X-ray (EDX) analyzer (FEI Company, Hillsboro, Oregon) based on a silicon detector and an S-UTW-Window operating at 30 kV acceleration voltage. The values of optical transmittance were measured in the wavelength range of 250–900 nm using an Ocean Optics spectrometer (Orlando, FL, USA) The dependence of the transmittance of optically transparent nanostructured NiO/Au and TiO_2_/Au films on hydrogen gas concentration in the range of 100 to 500 ppm was investigated. The experimental setup consisted of a gas flow chamber with an optical window, a light source (AvaLight-DH-S-BAL, Avantes) (Apeldoorn, The Netherlands), a USB4000 fiber optic spectrometer (Ocean Optics), optical fibers, and a computer with spectroscopy software (SpectraSuite 2.0 Ocean Optics) for transmission measurements (Figure 8). The target glass plates with the oxides were placed on a hot plate in the gas flow chamber. Using a temperature controller, the temperature of the hot plate was set to 300 °C and stabilized for 30 min. Then, a mixture of hydrogen and synthetic dry air was allowed to flow through the chamber at a flow rate of 200 mL/min. After the response stabilized (15 min), the optical spectra were acquired in the range of 300 to 900 nm. To evaluate the dependence of transmittance on hydrogen concentration at the point of highest response change, the transmittance values at 350 nm for the NiO/Au and 820 nm for the TiO_2_/Au were recorded, respectively.

## 3. Results and Discussions

### 3.1. Laser Conditions and Corresponding Surface Structures on Different Materials

LIPSS can be generated in a large variety of shapes and feature sizes depending on laser irradiation conditions. The dominant parameters are laser fluency (depends on laser power and focus diameter), number of pulses, number of overscans and selected materials.

**Figure 9:**
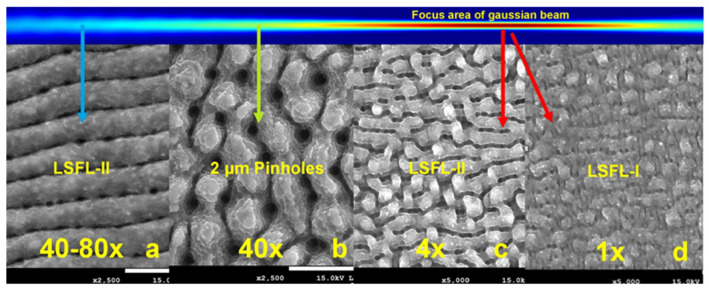
Metamorphosis of LIPSS depending on fluence and number of over-scans. (**c**,**d**) Highest fluence in the focal plane and lowest in (**a**). Sample position indicated by the colored arrows. At an intermediate fluence and 40 overscans pinholes are generated (**b**). The fluence is related to the sample position indicated by the colored arrows.

Figure 9 gives a visual impression of the evolution and transition between different types of LIPSS at the surface of an initial polished Si wafer. Directly in the focus plane (indicated by the red arrow) is the beam diameter smallest and the fluency highest at a given laser power. If the laser power is kept constant, the fluency can be reduced by changing the focus position relative to the specimen. Moving the focus out of the work plane (green and blue arrows) increases the beam diameter and reduces the fluency. In Figure 9c,d, the fluency is the same but the number of accumulated overscans is 4 times lower for the result in Figure 9d. Four accumulated scans in han in Figure 9c, are causing a change from LSFL-I ripple type to LSFL-II. In Figure 9a,b, the fluency is further reduced according to the focus position but the number of overscans increases to 40 and 80. This generates a structure with 2 µm pinholes and again transformation into the LSFL-II type, but a different shape compared to Figure 9c (see Figure 9a). We investigated the formation mechanism for the pinholes in [18]. The great variety of possible structures makes the femtosecond laser an ideal tool for surface functionalization. However, it needs experience with the specific laser setup, the used materials and the possible interference inside a certain process chain. A comprehensive library for orientation is now available in the literature. Here, we can give only a basic impression of possible applications and existing limits. There is definitely a great potential for LIPSS to tailor surface structures for a large field of applications, among them gas sensors. We faced limitations in the direct structuring of Si/SiO_2_ layer substrates and the electrical beak-down quality of thermal Si/SiO_2_ on laser-structured Si substrates. However, this led us to the LIPAA process and backside nanostructuring of glass substrates which can be used for electrical and optical sensor applications. The same process can be utilized for metal oxide nanoparticle fabrication and opens new opportunities for integration into our research portfolio. The LIPAA process utilizes the field enhancement features of nanoparticles in a backside ablation configuration to reduce the ablation threshold. In the following, we visualize this effect. One can also observe this in a standard front-side ablation regime. During the laser process, particles are generated. Such particles can cause scattering effects on the surface.

**Figure 10:**
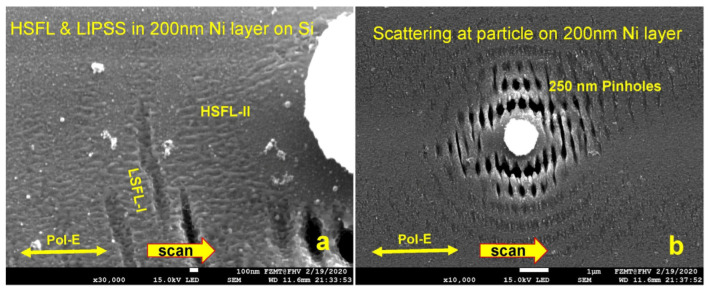
(**a**) HSFL-II formation in the area of low laser intensity and LSFL-I formation at high-intensity position. (**b**) Light field modulation and spatial enhancement by an oxide particle, pinhole formation close to the particle. This is partly reminiscent of the visualization of threshold lowering in the LIPAA process. No LIPSS at a greater distance to the particle observed.

Figure 10 shows such a scattering pattern on a sputtered 200 nm thick Ni layer on Si. The laser power was tuned just below the ablation threshold. The spatial field enhancement by the scattering particle shifted the laser power at some locations above the threshold and caused significant formation of nano-sized pinholes that can grow together and form 2–3 µm pinholes as shown in Figure 9b. In [18], we developed the means to suppress or promote pinhole growth by polarization switching. One can recognize in Figure 10a that two different types of periodic line structures began to evolve in the modulated light field. The LSFL-I type is perpendicular to the polarization of the laser and HSFL-II type is parallel to the polarization in the area with lower light intensity. On a glass substrate, we achieved with the LIPAA process uniform formation of 100 nm HSHL-I structures for sensor research. It shall become our next challenge to generate HSFLs and a sensitive nanoparticle film in situ in the same process. Figure 10b shows an enlarged area in the vicinity of the particle with about 250 nm in diameter pinholes. One can recognize in Figure 10a that two different types of periodic line structures began to evolve in the modulated light field.

### 3.2. Metal Oxide Nanoparticles on Glass and Their Optical Gas Responses

**Figure 11:**
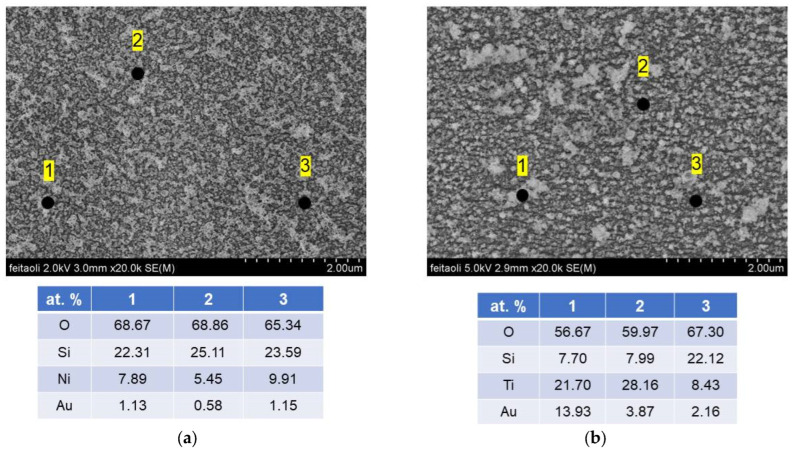
FE SEM images in detail and EDX analysis of selected elements of the target nanostructres for (**a**) Ni/Au and (**b**) Ti/Au sources.

**Figure 12:**
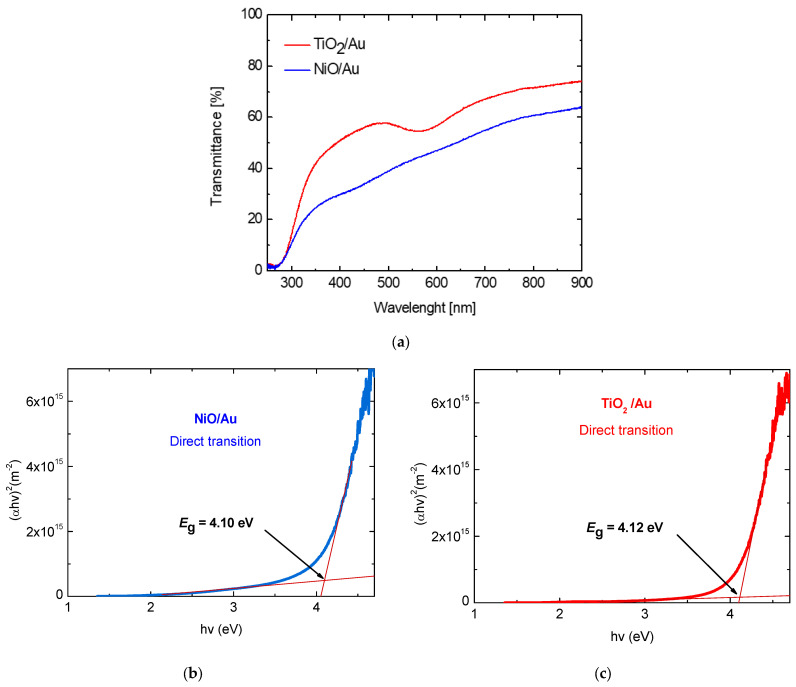
(**a**) Optical transmittance spectra and optical band gap values determined using the Tauc method for (**b**) NiO/Au and (**c**) TiO_2_/Au target nanostructures.

Figure 11 shows SEM images in detail of the target nanostructures for Ni/Au (Ti/Au) sources. The presence of individual elements of the examined samples was determined using EDX analysis. The EDX microanalysis performed during SEM observations on different points revealed that Ni, O, Au and Si are present in the Au/NiO layer in different concentrations (Figure 11a). Also, in the case of Au/TiO_2_, all elements, Ti, O, Au and Si, were recorded in the layer (Figure 11b). The investigations of the optical parameters allow us to obtain important information such as optical transmittance, absorption coefficient (α) and energy band gap (Eg) of prepared target nanostructured NiO/Au and TiO_2_/Au films. The values of optical transmittance were measured in the wavelength range of 300–900 nm using an OceanOptics spectrometer (Figure 12). It was found that all investigated nanostructured films are transparent and semiconducting. Using the Tauc method, the band gaps *E*_g_ were calculated and they are shown for NiO/Au and TiO_2_/Au target nanostructures in Figure 12.

**Figure 13:**
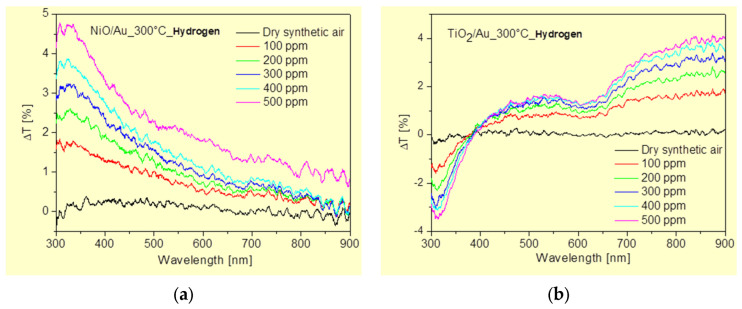
Changes in transmittance with the effect of H_2_ concentrations on (**a**) NiO/Au and (**b**) TiO_2_/Au target nanostructures.

Materials whose optical properties change reversibly by atmospheric gases are known, and these materials have considerable potential for use as optochemical sensors [20,21]. Such sensor elements could have several advantages over classic gas sensors that evaluate electrical parameters. Here, one can include more accurate evaluation as well as better selectivity due to recorded changes in the intensity, wavelength and polarization of the output optical signals. In order to identify the optical properties that can be suitable as a basis of optical answer for the sensor and its sensitivity, their optical transmittance under ambient atmosphere and H_2_ gas flow from 100 to 500 ppm at an optimal operation temperature of 300 °C were investigated. In Figure 13, the changes in transmittance of the samples show that by the effect of the hydrogen gas, there is a change in the transmittance measured. The adsorbed hydrogen is affecting the transmittance of the prepared nanostructured target NiO/Au and TiO_2_/Au films, respectively. We can observe small increasing changes in transmittance values with increasing hydrogen concentration in the gas chamber. We recorded a change in transmittance Δ*T* from 0.7% to 1.6% in the case of hydrogen detection with NiO and from 1.7% to 3.3% in hydrogen detection with TiO_2_ for the concentration from 100 to 500 ppm. Both of these dependencies are almost linear in the given ranges of hydrogen concentrations. From the given values, it is possible to calculate the sensitivity, which, in the case of NiO, represents a sensitivity value of 0.225%/100 ppm, and for TiO_2_ represents a sensitivity of 0.4%/100 ppm. We found almost twice the value of hydrogen sensitivity for nanostructured TiO_2_ compared to NiO. In the presence of reducing gases, including hydrogen, it was observed that thin layers such as NiO, Co_3_O_4_ and Mn_3_O_4_ show reversible changes in the visible near-IR band [22]. It is the combination of these metal oxides together with Au particles that can improve transmittance sensitivity due to the formation of hydrogen-based species on their surface. In the presence of reducing gases, including hydrogen, it was found that thin metal oxides exhibit reversible changes in optical parameters in the visible–IR band. In addition, the combination of these metal oxides and thin Au layers improves the sensitivity of the optical parameters to reducing gases. The observed changes in the transmittance of our samples show that by the effect of the hydrogen gas, there is a change in the measured transmittance. Recorded changes in the presence of H_2_ versus dry air in the maximum at 350 nm are caused by dipolar plasmon excitations of the Au/NiO composite film. These increased transmittance values can be correlated with an increase in the density of positive holes in the Au/NiO layer due to the increasing surface area with adsorbed ionized hydrogen particles.

## 4. Conclusions

We demonstrated the great potential of laser-induced periodic surface structures (LIPSSs) at various materials for surface functionalization and gas sensor applications. Electrical measurements indicate that the samples prepared by LIPSS generation on Si and subsequent thermal oxidation create locally spongy structures with low ohmic resistivity. Moreover, pre-surface-triggered nanoplasma explosions create nanobubble defects in the bulk close to the surface of LIPSS. Thermal oxidation encloses these nanobubbles inside the thin oxide layer causing a significantly reduced breakdown voltage. As a second solution, we produced HSFL on glass by backside ablation, providing 100 nm features with a high surface area and high resistivity. The prepared nanostructured target NiO/Au and TiO_2_/Au films showed a reversible change in the optical transmittance in the presence of H_2_ gas in concentration from 100 to 500 ppm. Almost twice the value of hydrogen sensitivity was recorded for the nanostructured target TiO_2_/Au compared to NiO/Au. We can state that such transparent materials offer the additional opportunity to produce optical gas sensors. In future research, we will investigate a possible integration of such optical sensors into polymer-based photonic waveguide devices.

## Data Availability

The original contributions presented in the study are included in the article, further inquiries can be directed to the corresponding author.
